# Antibacterial Activity of *Stenotrophomonas maltophilia* Endolysin P28 against both Gram-positive and Gram-negative Bacteria

**DOI:** 10.3389/fmicb.2015.01299

**Published:** 2015-11-24

**Authors:** Hongling Dong, Chaoyang Zhu, Jingyi Chen, Xing Ye, Yu-Ping Huang

**Affiliations:** ^1^College of Life Sciences, Wuhan UniversityWuhan, China; ^2^Hubei Provincial Cooperative Innovation Center of Industrial FermentationWuhan, China

**Keywords:** endolysin, *Stenotrophomonas maltophilia*, antibacterial activity, maltocin, EDTA

## Abstract

Maltocin P28 is a phage-tail like bacteriocin produced by *Stenotrophomonas maltophilia* P28. The ORF8 of maltocin P28 gene cluster is predicted to encode an endolysin and we name it endolysin P28. Sequence analysis revealed that it contains the lysozyme_like superfamily conserved domain. Endolysin P28 has the four consensus motifs as that of *Escherichia coli* phage lambda gpR. In this study, endolysin P28 was expressed in *E. coli* BL21 (DE3) and purified with a C-terminal oligo-histidine tag. The antibacterial activity of endolysin P28 increased as the temperature rose from 25 to 45°C. Thermostability assays showed that endolysin P28 was stable up to 50°C, while its residual activity was reduced by 55% after treatment at 70°C for 30 min. Acidity and high salinity could enhance its antibacterial activity. Endolysin P28 exhibited a broad antibacterial activity against 14 out of 16 tested Gram-positive and Gram-negative bacteria besides *S. maltophilia*. Moreover, it could effectively lyse intact Gram-negative bacteria in the absence of ethylenediaminetetraacetic acid as an outer membrane permeabilizer. Therefore, the characteristics of endolysin P28 make it a potential therapeutic agent against multi-drug-resistant pathogens.

## Introduction

*Stenotrophomonas* *maltophilia* is a Gram-negative bacillus and increasingly being recognized as an important nosocomial pathogen (Looney et al., 2009; Brooke, 2012). It can cause serious infections such as bacteremia, pneumonia, endocarditis, meningitis, urinary tract infections, skin and soft tissue infections in immunocompromised patients (Falagas et al., 2009; Sood et al., 2013; Hotta et al., 2014; Trignano et al., 2014; Guzoglu et al., 2015). The available therapeutic option for invasive *S. maltophilia* infection is limited, as this pathogen shows high levels of resistance to commonly used antibiotics (Sánchez, 2015). *S. maltophilia* not only exhibits intrinsic multidrug resistance but also can acquire antibiotic resistance during therapy like other pathogenic strains (Tan et al., 2008). Therefore, the novel treatment strategies and the effective antimicrobial agents are needed urgently to date for treatment of *S. maltophilia* infections.

Endolysins are enzymes encoded by phages at the end of their replication cycle to degrade the peptidoglycan of the host cell wall for cell lysis and release of the mature progeny phage particles (Loessner, 2005). Due to the absence of an outer membrane in Gram-positive bacteria, endolysins can easily access the peptidoglycan of cell wall and destroy these organisms when applied externally (Fischetti, 2010; Gilmer et al., 2013). When the endolysins work with Gram-negative bacteria, the chelating agents, like ethylenediaminetetraacetic acid (EDTA), are often used to increase the bacterial outer membrane permeability (Briers et al., 2011; Walmagh et al., 2013). Endolysins have been reported to be applied in medicine, control and detection of food-borne pathogens (Fischetti, 2008; Grandgirard et al., 2008; Coffey et al., 2010; Schmelcher et al., 2012). Endolysins can also be expressed by transgenic plants to prevent infection by phytopathogenic bacteria (Oey et al., 2009). Expanding databases of predicted proteins from the increasing number of sequenced and annotated bacterial genomes present a growing number of potential endolysins (Schmitz et al., 2010; Farris and Steinberg, 2014). As multidrug-resistant strains are becoming more prevalent, endolysins may be employed as novel alternatives to antibiotics (Bragg et al., 2014; Yang et al., 2014).

In the previous work, we have identified the maltocin P28 from *S. maltophilia* P28. The gene cluster of maltocin P28 is presumed to have 23 open reading frames (ORFs), and ORF8 is predicted to encode an endolysin (Liu et al., 2013). Here we designate this putative endolysin as endolysin P28. Sequence analysis revealed that the endolysin P28 contained the lysozyme-like superfamily conserved domain and its amino acid sequence had high identity with that of lambda phage gpR. In this study, we cloned the ORF8 into pET-26b(+) to express this endolysin gene in *Escherichia coli*. The recombinant endolysin P28 was purified and characterized. And the antibacterial activity of endolysin P28 against various Gram-negative and Gram-positive bacteria was also detected.

## Materials and Methods

### Bacterial Strains and Growth Conditions

*Stenotrophomonas maltophilia* P28 and *E. coli* BL21(DE3) were routinely cultivated in LB broth at 30°C. One hundred microgram per milliliter of ampicillin or 50 μg/mL kanamycin was added when necessary. Strains used for antimicrobial spectrum determination were all cultured in LB broth. The growth temperature was listed in **Table 1**. Most tested strains were taken from China Center for Type Culture Collection (CCTCC), the others were purchased from the American Type Culture Collection (ATCC) and *Aeromonas hydrophila* strain XS91-4-1 was kindly provided by Professor Aihua Li at Institute of Hydrobiology, Chinese Academy of Sciences.

**Table 1:** Antimicrobial activity of endolysin P28 against various species.

Strain	Species	Growth temperature (°C)	Antibacterial rate (%)
**Gram-positive bacteria**			
*Bacillus cereus*	CCTCC^a^ AB200055	30	>99
*Bacillus subtilis*	CCTCC AB93017	30	>99
*Listeria monocytogenes*	CCTCC AB209106	30	90.4
*Staphylococcus aureus*	CCTCC AB91053	37	>99
**Gram-negative bacteria**			
*Aeromonas hydrophila*	XS91-4-1	30	47.1
*Aeromonas salmonicida*	CCTCC AB98041	22	19.2
*Enterobacter aerogenes*	CCTCC AB91102	37	58.3
*Escherichia coli*	ATCC47076	37	60.3
*Klebsiella pneumoniae* ssp. *pneumoniae*	CCTCC AB2010163	37	>99
*Proteus vulgaris*	CCTCC AB91103	30	61.2
*Pseudomonas aeruginosa*	ATCC 15692	30	70.6
*Pseudomonas fluorescens*	CCTCC AB92001	30	0
*Pseudomonas putida*	ATCC12633	30	34.7
*Salmonella typhimurium*	CCTCC AB204062	37	0
*Shigella flexneri*	CCTCC AB200061	37	>99
*Xanthomonas citri* ssp. *malvacearum*	CCTCC AB96030	30	>99


*^a^CCTCC, China Centre for Type Culture Collection.*

### Plasmid Construction and Transformation

DNA manipulations were performed according to standard protocols (Sambrook, 1989). Genomic DNA of *S. maltophilia* P28 was extracted using the genomic DNA extraction kit (TIANGEN, China) and used as the template to amplify the ORF8 of maltocin P28 gene cluster. Plasmid DNA was obtained with TIANprep Mini Plasmid kit (TIANGEN, China).

### Cloning, Expression, and Purification of Endolysin P28

The ORF8 (putative endolysin gene) was amplified from the genomic DNA of *S. maltophilia* P28 by polymerase chain reaction (PCR). The PCR primers were endo-F (5′-CATATGACCGCCGCTGCAGCCAG-3′) and endo-R (5′-CTCGAGCTGCAGGGCTCCGCC-3′). Amplification was performed in PTC100 (BioRad, USA) with the following condition: 95°C for 5 min, 30 cycles of 94°C for 30 s, 55°C for 30 s, 72°C for 30 s, and the final extension with 72°C for 10 min. The PCR product was purified with Cycle-pure-Kit (OMEGA) and cloned into pDM19-T vector to confirm the sequence. Then the recombinant plasmid pDM19-T-ORF8 was digested with restriction enzymes *Nde*I and *Xho*I. The obtained 492-bp fragment was purified and inserted into pET-26b(+) by *Nde*I and *Xho*I, designating the recombinant plasmid pET-26b-ORF8. The *E. coli* BL21 (DE3) was transformed with plasmid pET-26b-ORF8. Transformants were cultivated in LB broth containing 50 μg/mL kanamycin at 37°C. Protein expression was induced by adding 1 mM isopropyl-β-D-thiogalactopyranoside (IPTG) at OD_600_ 0.5–0.8, followed by incubation for 6 h at 30°C. Bacterial cells were harvested at 4°C, suspended in binding buffer (0.5 M sodium chloride, 50 mM Tris-HCl, pH 7.5), and disrupted by sonication on ice. The lysates were centrifugated at 13,000 *g* for 10 min. After centrifugation, the supernatant was passed through a Ni-NTA column (Novagen). Purification of endolysin P28 was performed according to the manufacturer’s instructions. The identity and purity of the protein were confirmed by sodium dodecyl sulfate-polyacrylamide gel electrophoresis (SDS-PAGE). The gel was stained by Coomassie brilliant blue R-250 to visualize the bands. The purified endolysin P28 was stored at –20°C after the buffer was changed to the storage buffer (20 mM Tris-HCl, pH 7.0) by ultrafiltration.

### Determination of the Lytic Activity

The antibacterial activity of endolysin P28 was assessed by CFU reduction analysis as described previously (Rodriguez et al., 2011). Exponentially growing cells were centrifuged to discard the supernatant, and then the cell pellet was washed twice, resuspended in 20 mM Tris-HCl buffer (pH 7.0) and adjusted the OD_600_ to 0.5 ± 0.05. Purified endolysin (100 μL) was added to 300 μL cell suspension. The volume of reaction system was 400 μL. Generally the concentration of the endolysin added was adjusted to 2 mg/mL and the working concentration was 0.5 mg/mL. When the appropriate working concentration was tested, the concentration of the purified endolysin was changed while the reaction volumes of purified endolysin (100 μL) and cell suspension (300 μL) remained unchanged. After incubation for 1 h at 30°C, the mixture was serially diluted by 10-fold. To determine the survival rate, 100 μL 10-fold serial dilutions were plated on LB agar in triplicate, then colony forming units (CFUs) were counted after incubation at 30°C for 24 h. The number of CFUs between 30 and 300 on the spread plate was effectively counted. The control was performed by adding 100 μL 20 mM Tris-HCl buffer (pH 7.0) instead of endolysin P28. The antibacterial activity was expressed as the bacterial counts decrease. This value was calculated as the dead percentage referred to an untreated control. In this study, all the data were statistically analyzed by using the Origin8.1 program. Difference was investigated by the *T*-test at the 5% level. All experiments were repeated at least three times. The error bars represent the standard deviations.

In order to analyze the effect of EDTA, the above washed *S. maltophilia* culture (OD_600_ 0.5 ± 0.05) was incubated in 20 mM Tris-HCl buffer (pH 7.0) containing 0, 1 mM and 5 mM EDTA for 30 min. After centrifugation to remove the EDTA, the cell pellet was resuspended in 20 mM Tris-HCl buffer (pH 7.0). Then the purified endolysin was added and its antibacterial activity was determined as described above.

To evaluate the effect of pH on endolysin lytic activity, the endolysin (100 μL, 2.0 mg/mL) was added to 300 μL *S. maltophilia* cells suspended with a variety of buffers: 20 mM NaAc for pH 4.0 and pH 5.0, 20 mM Na_2_HPO_4_ -NaH_2_PO_4_ for pH 6.0, 20 mM Tris-HCl for pH 7.0, pH 8.0 and pH 9.0, Glycine-NaOH for pH 10.0–12.0. The control was carried out by adding 100 μL respective buffer instead of endolysin P28. After incubation for 1 h at 30°C, the CFUs of the mixture were determined by 10-fold dilution and the antibacterial activity was calculated as described above.

The influence of saline concentration on the lytic activity of endolysin was tested with NaCl concentration from 50 to 400 mM to cell suspension. One hundred microliter purified endolysin (2.5 mg/mL) was added to 300 μL exponentially growing cell (OD_600_ 0.5 ± 0.05), which was washed and resuspended in 20 mM Tris-HCl buffer (pH 7.0). And then 100 μL NaCl solution of different concentration was added to the mixture. The control was performed by adding 100 μL 20 mM Tris-HCl buffer (pH 7.0) instead of endolysin P28. After incubation for 1 h at 30°C, the CFUs were determined by 10-fold dilution and the antibacterial activity was calculated as described above.

In order to examine the effect of different temperatures on the enzymatic activity of endolysin P28, 100 μL purified endolysin (2.0 mg/mL) was added to 300 μL exponentially growing cell (OD_600_ 0.5 ± 0.05), which was washed and resuspended in 20 mM Tris-HCl buffer (pH 7.0). The control was performed by adding 100 μL 20 mM Tris-HCl buffer (pH 7.0) instead of endolysin P28. After incubation for 1 h at different temperatures (25–45°C), the CFUs were determined by 10-fold dilution and the antibacterial activity was calculated as described above.

Thermostability was tested by incubating endolysin P28 (2.0 mg/mL) in 30, 40, 50, 60, 70 for 30 min, and then 100 μL treated endolysin P28 was mixed with the above washed *S. maltophilia* cells (OD_600_ 0.5 ± 0.05). The control was performed by adding 100 μL 20 mM Tris-HCl buffer (pH 7.0) instead of endolysin P28. After incubation for 1 h at 30°C, the CFUs of the mixture were determined by 10-fold dilution and the antibacterial activity was calculated as described above.

## Results

### Sequence Analysis

**FIGURE 1:**
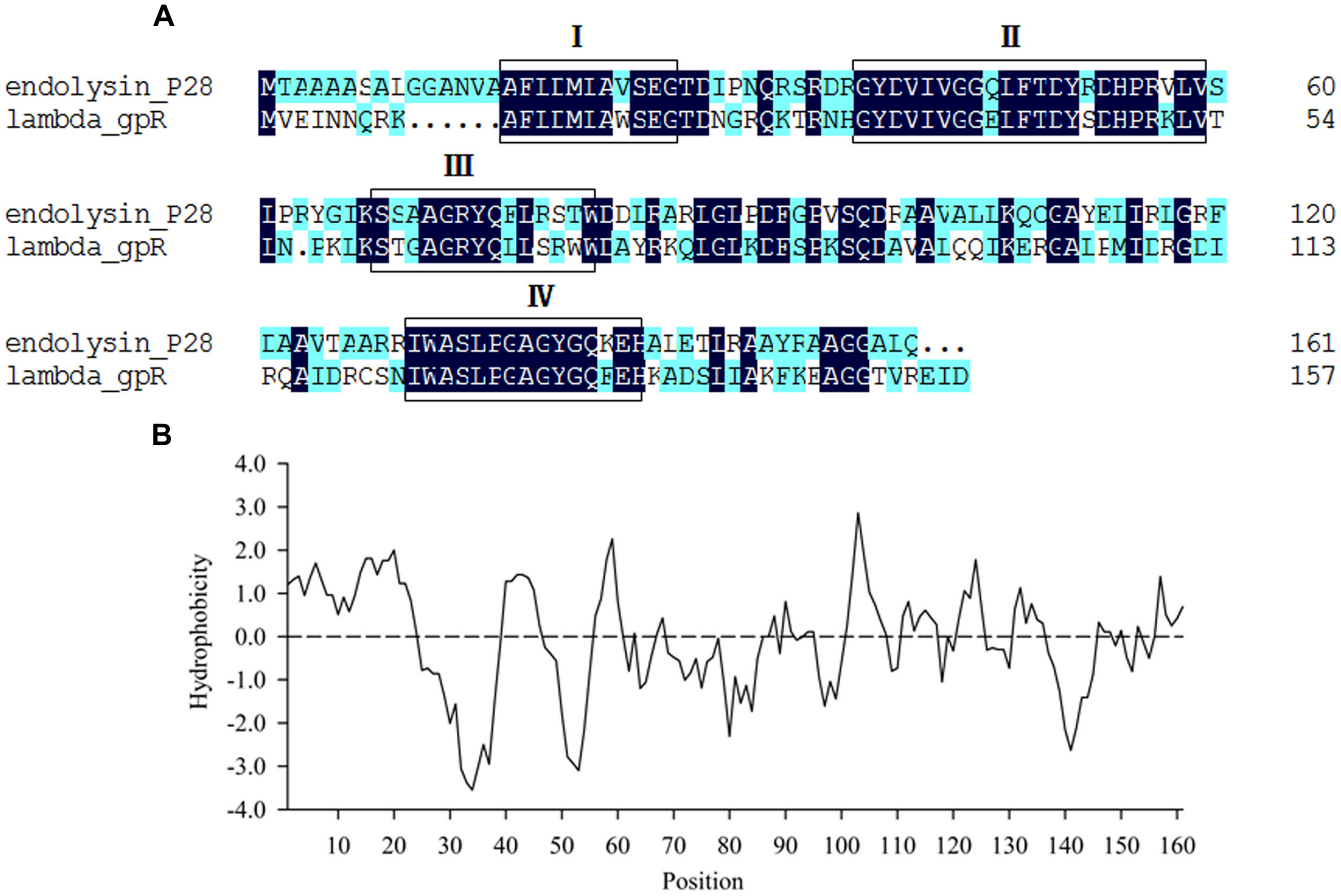
**Sequence analysis of endolysin P28.** **(A)** Comparison of amino acid sequences between endolysin P28 and lambda gpR, the boxes indicate the four consensus motifs. **(B)** Hydrophobicity analysis of endolysin P28.

Maltocin P28 is a novel phage tail-like bacteriocin produced by *S. maltophilia* P28 and has been characterized previously (Liu et al., 2013). The ORF8 of maltocin P28 gene cluster was predicted to encode an endolysin designated as endolysin P28. The deduced amino acid of endolysin P28 showed 48.48% identity with that of phage lambda gpR, which is the lambda lysozyme and function as a lytic transglycosylase. NCBI Blastp revealed that endolysin P28 included a conserved domain known as bacteriophage_lambda_lysozyme family. This family of lysozymes contains four consensus motifs (Blackburn and Clarke, 2001). Endolysin P28 has the same motifs, with the essential catalytic residue (Glu25) in motif I as lambda lysozyme gpR (Jespers et al., 1992) (**Figure 1A**). Compared with lambda gpR, endolysin P28 contained an N-terminal hydrophobic region (**Figure 1B**).

### Expression and Purification of Endolysin P28

**FIGURE 2:**
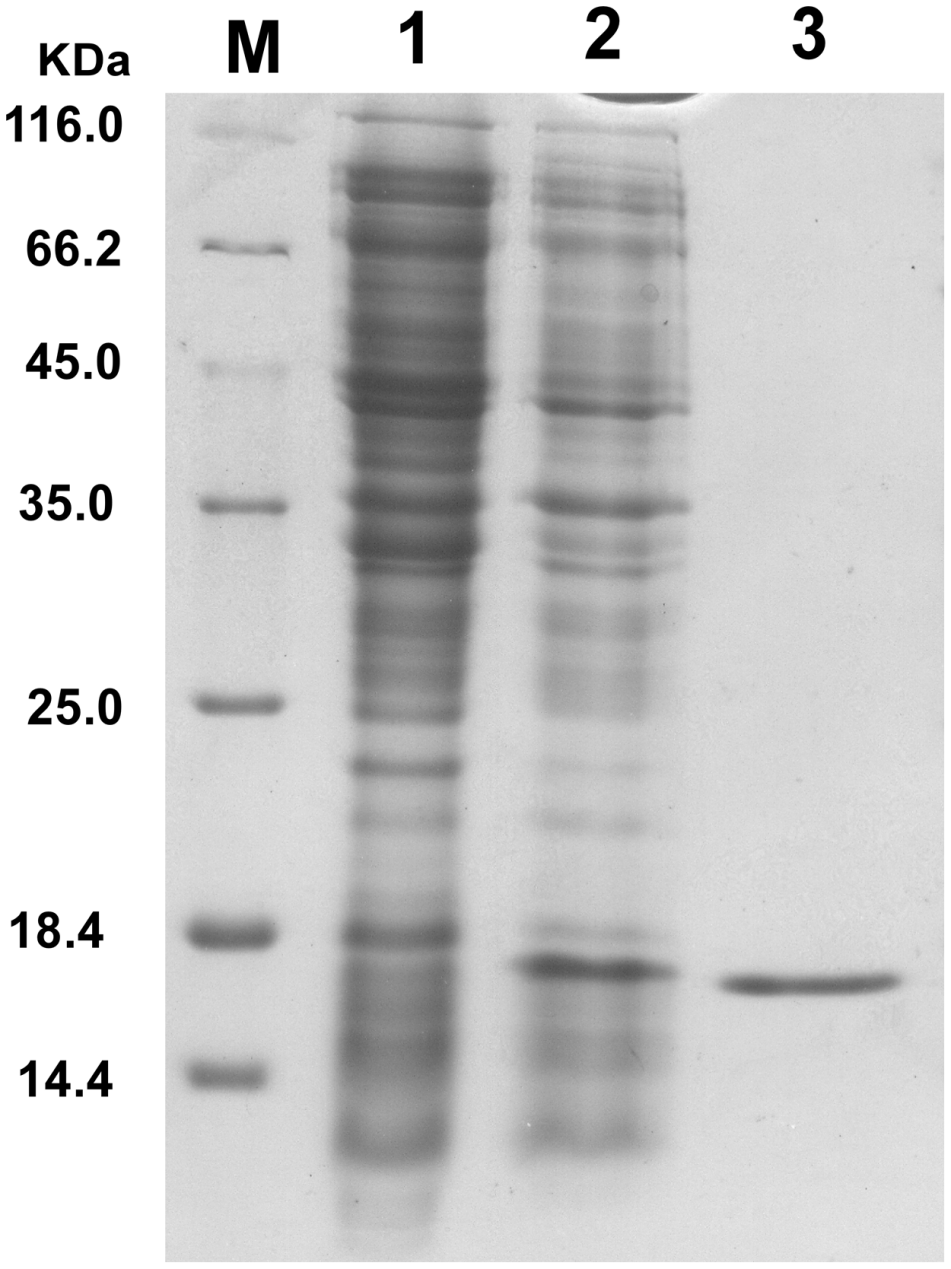
**Overproduction of endolysin P28 in *Escherichia coli* BL21 (DE3) cells.** Shown is SDS-PAGE (15%) of the cellular proteins from 80 μL cultures of bacteria. The gel was stained by Coomassie brilliant blue R-250 to visualize the bands. *E. coli* BL21 (DE3; pET-26b-ORF8) before induction (lane 1) and 6 h after induction (1 mM IPTG; lane 2). The overexpressed oligohistidine-tagged endolysin P28 was purified (lane 3). M, molecular masses of reference proteins (Thermo Scientific; in kilodaltons).

Endolysin P28 was overproduced as a C-terminal 6× His-tagged fusion protein which allowed purification by immobilized metal chelate affinity chromatography. The elution fractions were pooled and analyzed by 15% SDS-PAGE. A major protein band was observed which correlated well with the calculated mass of the endolysin P28 fusion protein (18.1 kDa; **Figure 2**). The concentration of purified endolysin P28 was determined according to the Bradford assay.

### Lytic Activity of Endolysin P28

**FIGURE 3:**
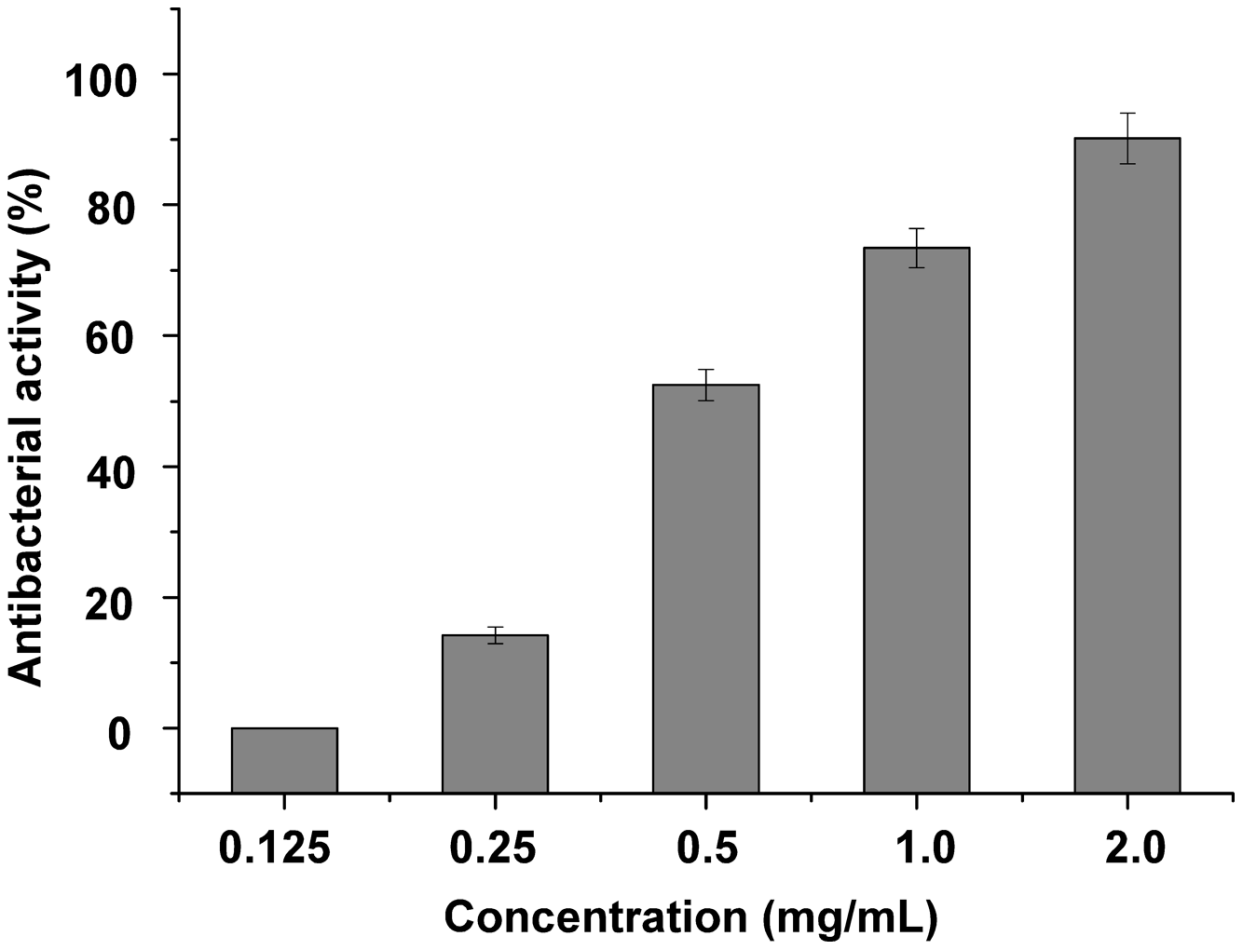
**Lytic activity of different concentration endolysin P28 against *Stenotrophomonas maltophilia* P28.** One hundred microliter purified endolysin P28 was mixed with 300 μL cell suspension (OD_600_ 0.5 ± 0.05). The concentration of the purified endolysin was changed while the reaction volumes of purified endolysin (100 μL) and cell suspension (300 μL) remained unchanged. After incubation for 1 h at 30°C, the CFUs of the mixture was determined and the antibacterial activity was calculated as described in the section “Materials and Methods”. The data were statistically analyzed by using the Origin8.1 program. All experiments were repeated at least three times. Mean values (±SD) are shown.

For antibacterial activity measurement, *S. maltophilia* P28 was used as substrate for the purified endolysin. The lytic activity of endolysin P28 was conducted by CFU reduction assay. The addition of 125 μg/mL endolysin did not change the viable numbers of *S. maltophilia*. When the endolysin concentration rose more than 125 μg/mL, its antibacterial activity enhanced as the concentration increased (**Figure 3**). When the endolysin concentration was 500 μg/mL, the CFU number reduced about a half. Thus the endolysin concentration of 500 μg/mL was used as the standard to perform the following antibacterial assays.

**FIGURE 4:**
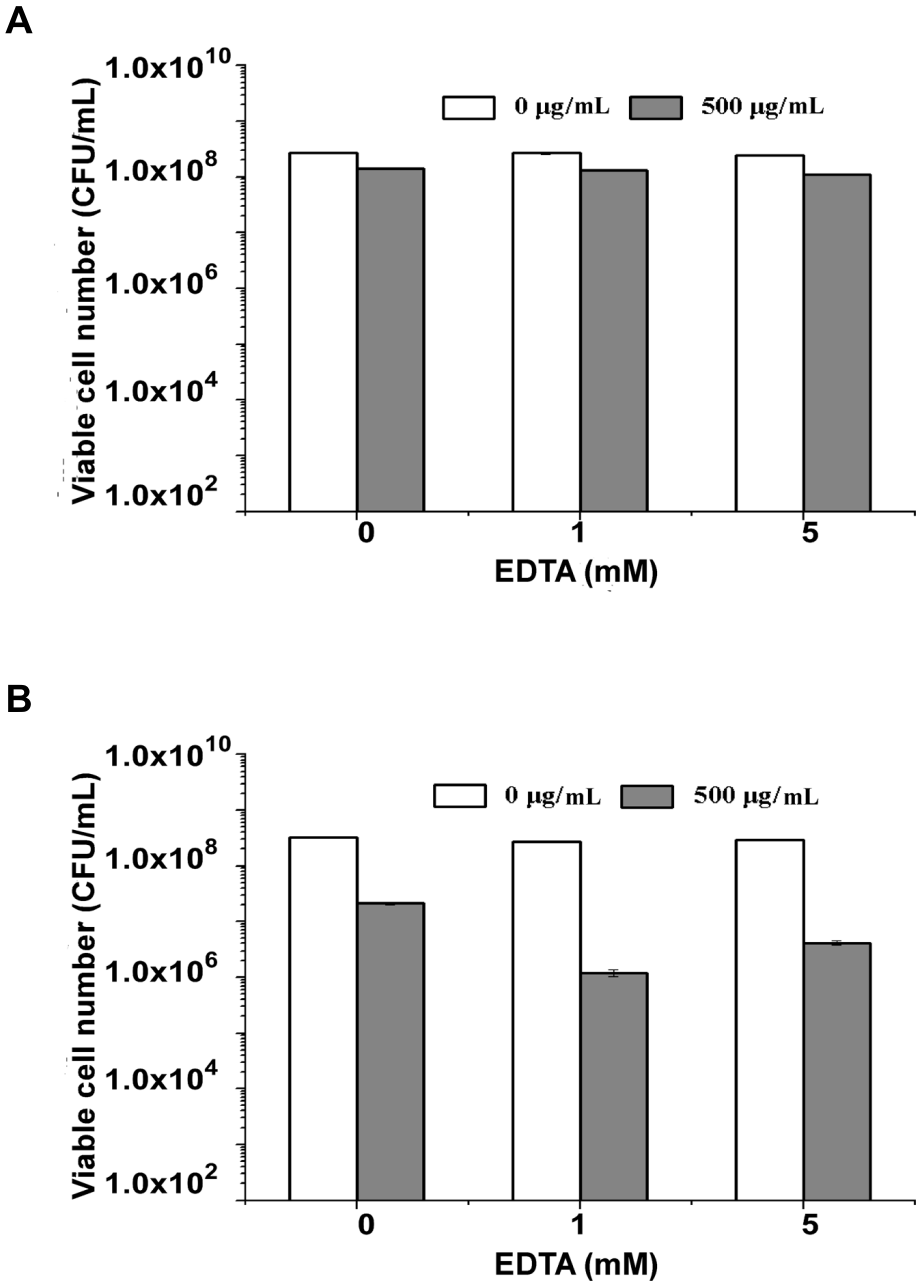
**Lytic activity of endolysin P28 over a range of ethylenediaminetetraacetic acid (EDTA).** The viable cell numbers (CFUs) of *S. maltophilia* P28 **(A)** and *P. aeruginosa* ATCC 15692 **(B)** after the addition of 500 μg endolysin P28 were examined. The cells were treated with different concentrations of EDTA (0, 1, 5 mM) for 30 min before the endolysin P28 was added. After incubation for 1 h at 30°C, the CFUs of the mixture was determined and the antibacterial activity was calculated as described in the section “Materials and Methods”. Error bars are the means ± standard deviation of three independent assays.

Gram-negative bacteria are more sensitive to endolysins after they are treated with EDTA to enhance the permeability of their outer membrane (Briers et al., 2011). To determine the effect of EDTA on the antimicrobial activity of endolysin P28, both *S. maltophilia* P28 and *Pseudomonas aeruginosa* ATCC15692 were treated with the different concentrations of EDTA for 30 min. The addition of EDTA led the cell lysis of *P. aeruginosa* ATCC15692 to significantly increase compared with buffer alone. The viable cell number of *P. aeruginosa* ATCC15692 was reduced more than 2 logs by addition of 1 mM EDTA, while the sensitivity of *S. maltophilia* P28 to endolysin was not affected by addition of EDTA (**Figure 4**). Thus, we did not add EDTA in the following experiments.

### Influence of Temperature, Salinity, and pH on Endolysin P28 Antibacterial Activity

**FIGURE 5:**
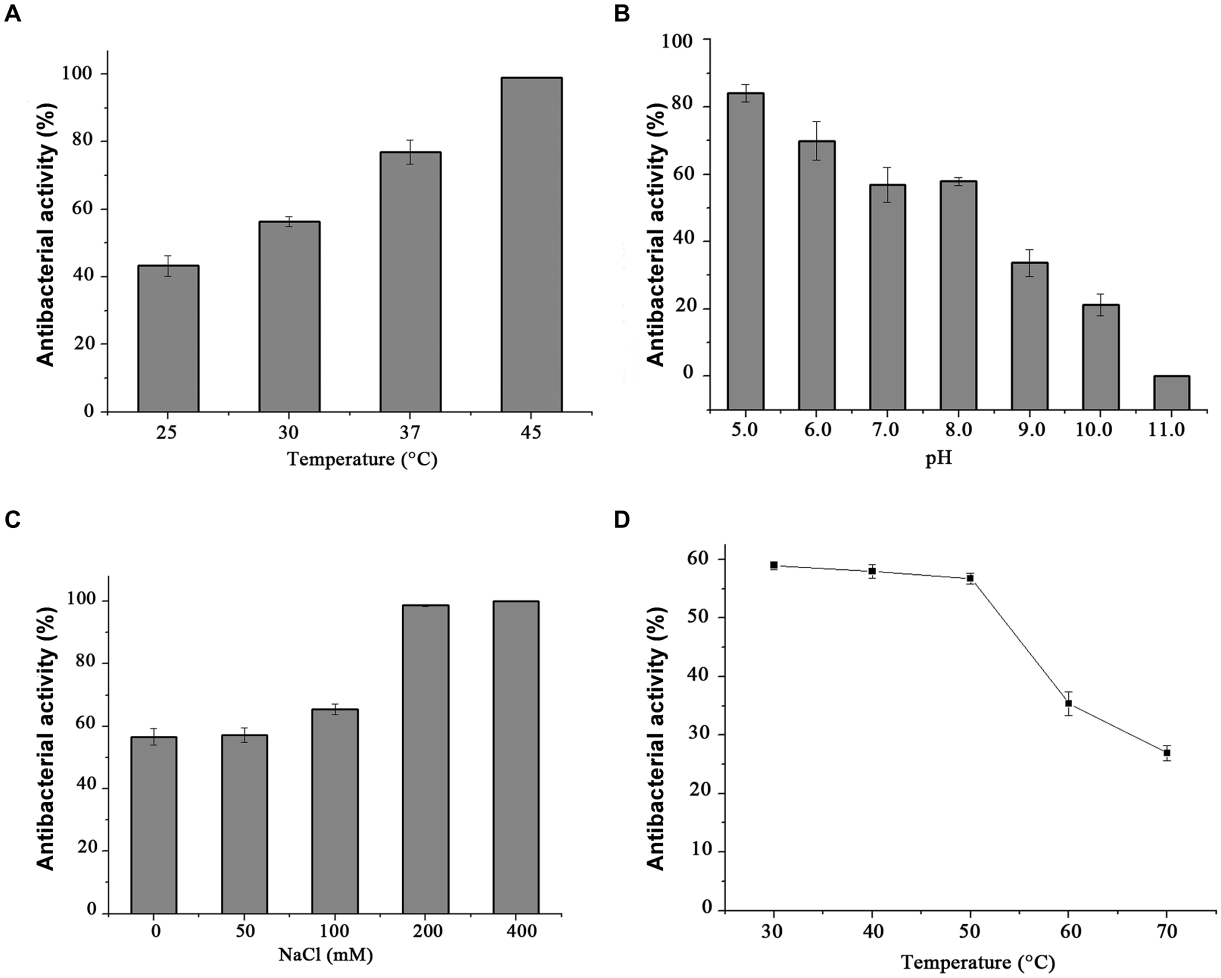
**Conditions for the enzymatic activity of the endolysin P28.** The antimicrobial activity was performed at different temperatures for 1 h in 20 mM Tris-HCl buffer, pH 7.0 **(A)**, and in buffers with different pH values for 1 h at 30°C **(B)**. The influence of saline concentration on the lytic activity of endolysin was tested with different NaCl concentration to cell suspension for 1 h in 20 mM Tris-HCl buffer, pH 7.0 at 30°C **(C)**. Thermostability of endolysin P28 was conducted by incubating endolysin P28 in different temperatures for 30 min, and then the residual activity was calculated as described in the CFU reduction assay **(D)**. Error bars are the means ± standard deviation of three independent assays.

Standard CFU reduction analysis was performed to analyze the effects of temperature, salinity, and pH on endolysin P28 antibacterial activity. The results showed that the antibacterial activity increased with the increase of temperature from 25 to 45°C (**Figure 5A**). And as the salt concentration increased from 100 mM to 200 mM, the antibacterial activity increased sharply. While the salt concentration up to 400 mM did not enhance the antibacterial activity further (**Figure 5C**). As for the effect of pH on endolysin P28, the result revealed that it had a relatively high lytic activity at a broad pH range of 5.0–8.0 and was ineffective when pH rose to 11.0 (**Figure 5B**). The thermal stability of endolysin P28 was characterized by challenges over a range of temperatures (30, 40, 50, 60, and 70°C). Residual activity of endolysin P28 was reduced 55% by following treatment at 70°C for 30 min (**Figure 5D**).

### Antibacterial Spectrum

**FIGURE 6:**
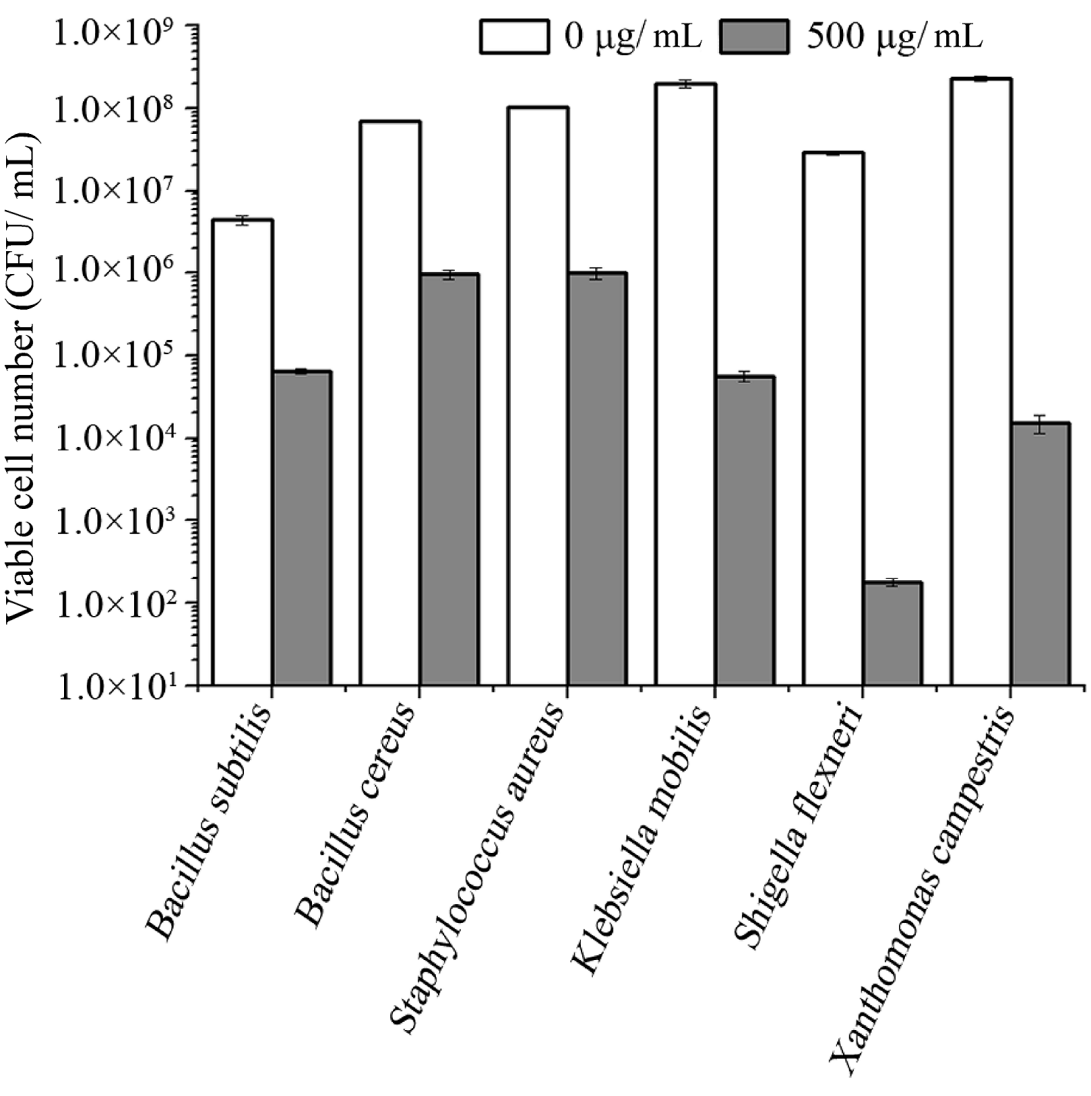
**Lytic activity of endolysin P28 against various Gram-positive and Gram-negative strains.** Various exponentially growing bacteria were washed twice and resuspended in 20 mM Tris-HCl buffer (pH 7.0). Three hundred microliter bacterial suspension (OD_600_ 0.5 ± 0.05) was mixed with 100 μL purified endolysin P28. After incubation for 1 h at 30°C, the CFUs of the mixture was determined and the antibacterial activity was calculated as described in the section “Materials and Methods”. Mean values (±SD) are shown.

To test the antibacterial spectrum of endolysin P28, we used four Gram-positive and twelve Gram-negative bacteria as substrates. Maltocin P28 only lyses *S. maltophilia* (Liu et al., 2013). Apparently endolysin P28 showed a broader antibacterial spectrum than maltocin P28 (**Table 1**). Endolysin P28 was able to lyse all of the tested Gram-positive bacteria and exhibited high lytic activity against three Gram-negative bacteria, *Klebsiella pneumoniae* ssp. *pneumoniae*, *Shigella flexneri*, *Xanthomonas citri* ssp. *malvacearum* (**Figure 6**). However, no activity was detected against *P. fluorescens* and *Salmonella typhimurium*.

## Discussion

The last decade has witnessed an increasing emergence of drug-resistant pathogens. Thus it is important to develop new alternatives to antibiotics. Phage endolysins have been reported to have good potential as a therapeutic agent for bacterial infections (Schmelcher et al., 2012). When added externally, they can cause a hole in the cell wall of the target bacterium through peptidoglycan digestion exerting a lethal effect (Fischetti, 2010). In general, exogenous endolysin is highly active against many Gram-positive species but ineffective against Gram-negative bacteria. It is probably due to the outer membrane existing in Gram-negative bacteria, which is impermeable to macromolecules and makes a physical barrier for endolysin to access the peptidoglycan layer (During et al., 1999; Fischetti, 2010).

Most endolysins from Gram-negative bacteriophage reported have only a catalytic domain. Some of them have a modular structure composed of a C-terminal catalytic domain and N-terminal cell wall binding domain (Briers et al., 2007; Fokine et al., 2008; Fischetti, 2010; Callewaert et al., 2011; Walmagh et al., 2013). Sequence analysis of endolysin P28 found no N-terminal cell wall binding domain or specific catalytic domain, rather it contained an N-terminal hydrophobic region (**Figure 1B**), which formed a putative helix domain. Some endolysins from phages of Gram-negative bacteria can destroy bacterial cells by means of a mechanism completely independent of their enzymatic activity (During et al., 1999; Orito et al., 2004). In these cases, it was found that helix-forming amphipathic peptides containing basic amino acid residues seem to interact with negatively charged membrane elements, such as lipopolysaccharide in Gram-negative bacteria (During et al., 1999; Lim et al., 2014). Therefore, we proposed that the N-terminal hydrophobic region of endolysin P28 should have the same function. Endolysin P28 showed a hydrolysis activity against peptidoglycan of *E. coli* and *Bacillus subtilis* through the turbidity reduction assay (data not shown). In addition to the four consensus motifs between endolysin P28 and lambda gpR, the catalytic residue Glu19 in lambda gpR playing an crucial role in the interaction of the enzyme with the GlcNAc of the peptidoglycan backbone is conserved in endolysin P28 (Glu25). So, further studies regarding the functions of N-terminal helix domain and Glu25 of endolysin P28 should be performed in the future.

Endolysin P28 had a good thermal stability, and showed a broad lytic spectrum against 14 out of 16 tested bacteria including medically important genera *Aeromonas*, *Bacillus*, *Escherichia*, *Klebsiella*, *Listeria*, *Pseudomonas, Proteus*, *Salmonella*, *Shigella*, *Staphylococcus*, and *Xanthomonas*. Since all the Gram-negative strains tested in this study are Gammaproteobacteria, further investigation into the effect of endolysin P28 on other bacterial species outside the two clusters of Gram-positive bacteria and Gammaproteobacteria is being carried out. Endolysin P28 could not lyse *S. typhimurium* and *P. fluorescens*. Gram-negative pathogens were protected by the presence of an outer membrane, preventing the entry of endolysins into the cell and reach the peptidoglycan (Nikaido, 2003). EDTA is a well-known used outer membrane permeabilizer that acts as a chelator by removing stabilizing cations from the outer membrane (Oliveira et al., 2014). In this study, we also found that EDTA could enhance the activity of endolysin P28 to *P. aeruginosa*. However, the tested strains were not treated by any outer membrane permeabilizer agent when the spectrum of endolysin P28 was detected. Thus, the reason for endolysin P28 ineffective against some strains may be the endolysin P28 could not pass through outer member and reach their peptidoglycans. Several *Salmonella* phage endolysins, such as Lys68, SPN1S, and SPN9CC, have been characterized (Lim et al., 2012, 2014; Oliveira et al., 2014). They have a wide spectrum of antibacterial activity against Gram-negative bacteria but no effect on the Gram-positive bacteria tested. More strains should be detected to ascertain how the antibacterial spectrum of endolysin is associated with the bacterial characteristics.

As *S. maltophilia* P28 is EDTA resistant, we have no wonder that EDTA had no effect on the sensitivity of *S. maltophilia* P28 to endolysin P28. However, it is important to note that endolysin P28 has antibacterial activity against other Gram-negative bacteria without EDTA treatment. To our knowledge, few endolysins and engineered phage lytic enzymes have been reported to have this property (Lai et al., 2011; Lukacik et al., 2012, 2013; Schmelcher et al., 2012). The antibacterial rate of endolysin P28 was more than 99% against six tested strains, which were three Gram-positive and three Gram-negative strains. The viable cell numbers of three Gram-positive strains were reduced about 2 logs, while the viable cell numbers of three Gram-negative strains were reduced more than 3 logs, especially the viable cell number of *S. flexneri* was reduced more than 5 logs (**Figure 6**). These results suggested that endolysin P28 is a potential therapeutic agent against both Gram-positive and Gram-negative pathogenic strains.

## Conflict of Interest Statement

The authors declare that the research was conducted in the absence of any commercial or financial relationships that could be construed as a potential conflict of interest.
